# Mixed Methods Evaluation of the Impact of Allied Health – Translating Research into Practice (AH-TRIP) Program on the Knowledge Translation Capacity of the Allied Health Workforce

**DOI:** 10.34172/ijhpm.8910

**Published:** 2025-10-27

**Authors:** Adrienne M. Young, Alita Rushton, Ashley Cameron, Nina Meloncelli, Shelley A. Wilkinson, Rachelle Pitt, Kathryn McFarlane, Katrina L. Campbell, Gillian Harvey, Ingrid J. Hickman

**Affiliations:** ^1^Centre for Health Services Research, Faculty of Medicine, The University of Queensland, Brisbane, QLD, Australia.; ^2^Dietetics and Food Services, Royal Brisbane and Women’s Hospital, Brisbane, QLD, Australia.; ^3^School of Health Sciences and Social Work, Griffith University, Gold Coast, QLD, Australia.; ^4^Office of the Chief Allied Health Officer, Queensland Health, Brisbane, QLD, Australia.; ^5^Healthcare Excellence and Innovation, Metro North Hospital and Health Service, Brisbane, QLD, Australia.; ^6^Office of the Chief Allied Health Practitioner, Metro North Allied Health, Brisbane, QLD, Australia.; ^7^Department of Obstetric Medicine, Mater Mothers’ Hospitals, Brisbane, QLD, Australia.; ^8^School of Pharmacy, Faculty of Health and Behavioural Sciences, The University of Queensland, Brisbane, QLD, Australia.; ^9^Allied Health, Cairns and Hinterland Hospital and Health Service, Cairns, QLD, Australia.; ^10^Caring Futures Institute, College of Nursing and Health Sciences, Flinders University, Adelaide, SA, Australia.; ^11^ULTRA Team, Clinical Trial Capability, Centre for Clinical Research, The University of Queensland, Brisbane, QLD, Australia.

**Keywords:** Health Workforce, Evidence-Based Practice, Implementation Science, Knowledge Translation, Capacity Building, Mentoring

## Abstract

**Background:**

Knowledge translation (KT) in healthcare is a complex process. Building the KT capacity of the workforce is fundamental to closing the gaps between research and evidence-based practice. This evaluation aimed to describe the impact of a KT capacity building program (Allied Health Translating Research into Practice, AH-TRIP) on allied health professionals and health services and systems, with the secondary aim of identifying barriers and enablers to program impact.

**Methods:**

Multi methods evaluation using online surveys and semi-structured interviews with 20 program participants and their managers. The interview guide was underpinned by the Framework to Assess the Impact from Translational health research (FAIT). Deductive content analysis was used to categorise impact against FAIT, with barriers and enablers mapped to an implementation framework.

**Results:**

Six domains of impact were identified: Individual Capacity Building, Workforce Capacity Building, Enhanced Networks, Clinical Practice Change, Patient/Community Benefits, and Economic Benefits. Enablers of impact were program design (flexibility, access to mentors, funding opportunities), local contexts supporting research (manager support, access to local experts), and previous exposure to KT. Consistent barriers included a lack of clinician time and confidence in KT, staff turnover (particularly in regional/rural areas), lack of organisational research culture, and short-term funding cycles.

**Conclusion:**

Using FAIT methodology, we have demonstrated significant impact achieved by a KT capacity building program for individual health professionals and the broader allied health workforce and health services and systems. Impact could be further optimised by strategies targeted at managers to create supportive contexts for KT through improving research literacy in health decision and policy contexts and innovative workforce planning.

## Background

**Key Messages**

**Implications for policy makers**

- Building knowledge translation (KT) capacity in the health workforce requires multiple approaches including accessible training, mentoring and support and a showcase and celebration of efforts.
- Engaging with KT capacity building generates broad ranging benefits above that of an individual’s development including clinical practice change, broader workforce capacity building and networks, potential health service cost savings and benefits for the community accessing healthcare.
- Embedding skill development into leadership programs and offering small “start-up” funding opportunities may enhance outcomes.
- Engagement with KT researchers and experts appears to be key to success for capacity building impacts.
- Supporting uptake and impact of KT capacity building in regional and rural areas requires targeted strategies to overcome barriers related to the rural health workforce.

**Implications for the public**

Keeping health service delivery up to date with the latest research evidence relies on having a skilled health workforce that is supported to understand and apply new information. The development of a program that provides training to build skills and confidence in health workers to bring research findings into everyday practice was implemented across an Australian state health service. The program involved online training, mentoring and support and showcasing knowledge translation (KT) efforts across hospital districts. Investment in KT skills and confidence in the health workforce created broad reaching benefits not only for the individual participating but for the broader workforce and their networks. People who took part in the program changed how they practice to better match the latest evidence, benefitting the community by providing better quality care from those health services.

Across the health system, there are recognised gaps between knowledge creation in research settings and the implementation of evidence-based practice. Whilst small scale, site-specific improvements can be achieved, system-wide transformations are challenging due to success often being dependent on local context, culture, priorities and resources.^1^ When health systems fail to effectively use evidence to inform care, the resulting inefficiencies negatively impact health outcomes.^2^ Knowledge translation (KT) is the process of implementing evidence into clinical practice with the goal of providing evidence-based care,^3^ and is a key strategy to improve health outcomes and efficiency^2^ and reduce research waste.^4^ KT is a complex process and fundamental to embedding KT activities into clinical practice is the KT capacity of the people who work in those settings.^5^ There is emerging evidence that KT training and mentoring in the health workforce improves self-efficacy, confidence and KT use among participants.^6-8^

**Figure 1:**
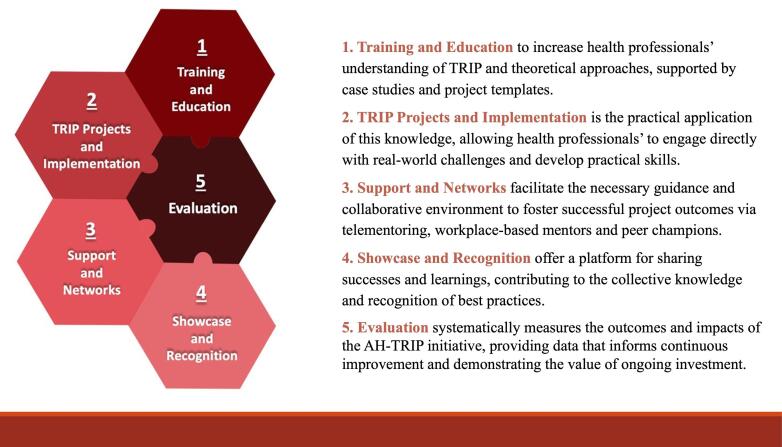
Overview of the Allied Health Translating Research into Practice Initiative. Abbreviation: AH-TRIP, Allied Health Translating Research into Practice. Note: TRIP is used as a synonym for knowledge translation.

 The healthcare workforce is diverse with variation in capacity and opportunity for KT,^9-11^ making it challenging to develop strategies and programs to address gaps in KT skills and confidence. The “Allied Health Translating Research into Practice” (AH-TRIP) initiative is a freely accessible multimodal KT capacity building program for front-line allied health professionals^12^ designed to address an established workforce need^9,10^ (Figure 1). Within three years of implementation, the program was adopted by multiple public hospitals and health services, with high levels of engagement (with nearly 1000 participants in the first three years),^12^ high participant satisfaction^12,13^ and measurable improvements in confidence in KT.^13^ To date, very few KT capacity building initiatives have measured longer-term impact beyond knowledge, self-efficacy and project progress.^14^ There is a need to determine what impacts are generated beyond these immediate outcomes, and how to optimise these impacts to maximise return on investment and provide a business case for adoption elsewhere. Multi methods evaluation of these programs using an impact evaluation framework may assist in clarifying the different domains of benefit achieved by such programs,^15-17^ and support decision-making about their implementation and ongoing investment.

 The aim of this evaluation was to describe the impact of a KT capacity building program on allied health professionals and health services and systems through the application of the Framework to Assess the Impact from Translational health research (FAIT), with a secondary aim of identifying barriers and enablers to the program achieving impact.

## Methods

###  Study Design and Theoretical Approaches

 This multi methods evaluation was underpinned by FAIT,^18^ a framework developed to capture research impact across multiple levels that may be useful to policy-makers, funders, researchers, and healthcare workers^18^ (overview provided in Table 1). In the context of FAIT, research impact is defined as the demonstrable benefits that research has for human health, quality of life and society beyond traditional academic outcomes.^18^ Though developed to be applied prospectively, FAIT has been applied retrospectively to evaluate the impact of research programs and centres,^19,20^ and was applied retrospectively in the present evaluation. In line with FAIT, both quantitative and qualitative data were used concurrently in this multi methods evaluation. Narratives were constructed according to FAIT scorecard templates. The integrated Promoting Action on Research Implementation in Health Services (i-PARIHS) framework^21^ was also applied to identify barriers and enablers to achieving impact. This framework was selected due to its consideration of factors at multiple levels related to the program itself (innovation), the people involved (recipients), the context at multiple levels, and how the implementation of the program was facilitated (Table 1). The Mixed Methods Reporting in Rehabilitation & Health Sciences checklist was used in the preparation of this manuscript^22^ (Supplementary file 1, Table S1).

**Table 1:** Overview of Frameworks Applied in This Evaluation

**FAIT**^18^	
Purpose	Designed as a prospective tool to capture processes, outcomes and impacts generated across the spectrum of health research
Overview	Combines three commonly used approaches to impact assessment: modified Payback method, economic analysis and narrative case studies
Domains	Domains of benefit (usually quantitative data): advance knowledge; clinical implementation; community benefit; legislation and policy; economic impact Social return on investment: cost benefit analysis Case studies (explain pathway to impact and bring together qualitative and quantitative data)
Use in AH-TRIP evaluation	Informed interview questions; domains of benefit used to code interview data; data synthesised into narrative case studies
**i-PARIHS**^21^	
Purpose	Theoretical framework to plan, guide and evaluate the implementation of evidence-based healthcare interventions
Summary	Theorises that successful implementation results from the facilitation of an innovation with the recipients in their context
Domains and example constructs	Facilitation: role and strategies that activate implementation by assessing and responding to characteristics of the innovation and the recipients within their contextual setting Innovation: eg, knowledge source, clarity, degree of fit, usability Recipients: eg, motivation; values and beliefs; goals; skills and knowledge; time, resources, support Context: (*a*) Local: eg, leadership support; culture; experience of change; (*b*) Organisation: eg, leadership and senior management support; culture; structure and systems; (*c*) Health system: eg, policy drivers and priorities; regulatory frameworks; networks
Use in AH-TRIP evaluation	Constructs used to code barriers and enablers from interview data

Abbreviations: FAIT, Framework to Assess the Impact from Translational health research; i-PARIHS, integrated Promoting Action on Research Implementation in Health Services; AH-TRIP, Allied Health Translating Research into Practice.

###  Setting

 The AH-TRIP program was designed in 2019 and has been implemented across public hospitals and health services in Queensland, Australia.^12^ This encompasses acute, sub-acute and community health services spanning an area of more than 1.8 million km^2^ and services a population of around 5.2 million people.^23^ The program consists of five pillars: (1) training and education, (2) project implementation, (3) support and networks, (4) showcase and recognition, and (5) evaluation^12^ (Figure 1). The program is voluntary, free of charge and targets (although not limited to) front-line allied health professionals, defined as health professionals from non-medical and non-nursing disciplines such as dietitians/nutritionists, occupational therapists, pharmacists, physiotherapists, psychologists, medical radiation therapists, social workers, and speech pathologists.^12^

###  Participants

 Allied health professionals were invited to participate in this evaluation if they had engaged with AH-TRIP between 2019 and 2021 in any of the following ways: received AH-TRIP support and telementoring, showcase presenters and/or recipients of locally funded AH-TRIP start-up grants. Individuals were identified via the AH-TRIP program manager records and were invited to participate through a personalised email from the centralised AH-TRIP email address. The email provided a comprehensive explanation of the evaluation’s objectives. A reminder email was sent two weeks after the initial invitation to encourage participation. Purposive sampling was used with the goal of achieving diversity across the sample related to allied health discipline, gender and health service location (ie, metropolitan, regional, rural, and remote areas) and to adequately reach information power.^24^

 To gain a wider perspective on the impact of AH-TRIP within the hospital and health service, each participant was then invited to identify and make initial contact with their manager (or other person who supported their AH-TRIP participation) about also participating in the evaluation. All nominated managers were contacted and invited to participate.

###  Data Collection

 Data were collected from participants using a semi-structured interview, an online questionnaire and available reports relevant to the KT project. The interview guide was developed by the authors (Supplementary file 2). Open ended questions were used to capture impact broadly, with prompts aligned to the FAIT domains and in consideration of other suggested impact indicators.^18,25^ The questions were piloted with the first recruited participant, which confirmed that all relevant questions were included; therefore, the data from the pilot interview was retained in the study. Interviews were conducted via Microsoft Teams by a single researcher not previously involved in AH-TRIP delivery or evaluation (AR, experience working in health services as an allied health assistant, research qualifications and experience with undertaking semi-structured interviews). Interview duration ranged from 17 to 42 minutes duration (mean: 28 minutes). Interview transcripts produced by Microsoft Teams were reviewed against audio recordings to maintain accuracy and stored as de-identified files to maintain confidentiality. A pre-interview questionnaire (Supplementary file 1, Table S2) was used to collect quantitative impact data (number of publications, presentations, project outcomes) and participant demographics (gender, geographical location, discipline, years of experience) prior to the interview commencing, which was cross-checked during the interview. Participants were also encouraged to provide copies of any reports that included data relevant to impact (eg, end of project reports providing data on clinical practice change, patient outcomes or cost-benefit analysis); where participants reported publishing findings in peer-reviewed manuscripts these were located and relevant data extracted.

###  Data Analysis

 Data were extracted from all data sources to construct a summary for each participant. These summaries aligned with FAIT and included Domains of Benefit (data from interview, questionnaire, reports/publications), Economic Analysis (interview, reports/publications), and Narrative (interview) components. Quantitative data from questionnaires and reports/publications were analysed descriptively and presented within each participant summary. Qualitative interview data were analysed using deductive content analysis,^26^ with first-round coding undertaken by the interviewer (AR) against a coding framework aligned to FAIT. All participant summaries were cross-checked against interview transcripts, questionnaires and reports/publications by a second researcher (AY). Selected participant summaries and associated de-identified transcripts were then shared with the whole research team for discussion of findings. Through this process, the Domains of Benefit were adapted, with the decision made to remove the “advance knowledge” domain (not considered relevant to AH-TRIP as it relates to generating new research knowledge rather than implementing knowledge) and add other domains related to capacity building and networks. These new domains were used in second-round coding (completed by AR and AY), data synthesis and interpretation, with metric categories identified for each domain and summary data provided as evidence for each, captured as counts for evidence from any source including interviews, surveys, published manuscripts or unpublished reports; (yes or no). Definitions for each construct were prospectively agreed, and counts were checked by two researchers (AY and AR). A Narrative was developed for each participant by AY and AR to describe the pathway to impact (involvement with AH-TRIP components and KT project), key example/s of impact (supported by quotes) and any barriers and enablers to impact experienced by the participant. The completed participant summaries (including Domains of Benefit, Economic Analysis and Narrative) were returned to each participant to check accuracy and interpretation; no changes were requested.

 The i-PARIHS framework^21^ was used as an analytical framework to identify the factors influencing the impact of AH-TRIP (ie, barriers and enablers to impact). Deductive content analysis involved a single researcher (AY) coding the interview data against the core i-PARIHS constructs (Recipients, Innovation, the Local, Organisation and External Contexts, Facilitation), mapping each factor to the Domains of Benefit and highlighting the barriers and/or enablers to impact within each domain. Findings were reviewed by a second researcher (AR).

## Results

 From 31 allied health professionals who had registered interest in participating, 20 were purposively selected for inclusion. Of the nine managers who were nominated by participants and invited to participate, five consented to participate.

 Characteristics of participants are displayed in Table 2. All AH-TRIP participants had implemented a KT project and had been involved with AH-TRIP as a showcase presenter (n = 9), received AH-TRIP mentoring and support (via telementoring program, n = 7; or one-on-one mentoring, n = 4), or received an AH-TRIP grant (project start-up grant, n = 3; bursary to attend additional external KT training, n = 3), with five participants involved in more than one part of AH-TRIP. Across the 20 participants, 11 KT projects were completed at the time of the interview, with seven projects still underway or ongoing. One project was on hold due to the COVID-19 pandemic, and one participant had moved to a new position and was unsure of the status of the project.

**Table 2:** Characteristics of Allied Health Translating Research into Practice Participants and Their Managers

		**AH-TRIP Participant (n = 20)** **No. (%)**	**Manager** **(n = 5)** **No. (%)**
Facility location	Metropolitan area	16 (80)	4 (80)
	Regional area	3 (15)	1 (20)
	Rural area	1 (5)	0 (0)
Allied health discipline	Nutrition & dietetics	8 (40)	1 (20)
	Occupational therapy	4 (20)	1 (20)
	Physiotherapy	6 (30)	2 (40)
	Social work	1 (5)	0 (0)
	Speech pathology	1 (5)	0 (0)
	Allied health director	0 (0)	1 (20)
Years of experience^a^	2-5 years	1 (5)	0 (0)
	5-10 years	2 (10)	0 (0)
	10-15 years	6 (30)	1 (20)
	15-20 years	5 (25)	1 (20)
	20+ years	5 (25)	3 (60)
Gender	Female	18 (90)	5 (100)
	Male	2 (10)	0 (0)
Role	Clinician	16 (80)	0 (0)
	Clinical educator	1 (5)	0 (0)
	Research fellow	1 (5)	1 (20)
	Team leader	1 (5)	2 (40)
	Discipline/allied health director	1 (5)	2 (40)

Abbreviation: AH-TRIP, Allied Health Translating Research into Practice. ^a^ Not answered: AH-TRIP participant n = 1.

###  Domains of Benefit (Modified Payback)

 Impact was identified across sixDomains of Benefit: Individual Capacity Building, Workforce Capacity Building, Enhanced Networks, Clinical Practice Change, Patient/Community Benefits, and Economic Benefits. These are summarised in Table 3.

**Table 3:** Summary of AH-TRIP Impact: Domains of Benefit (Modified Payback Method of Assessment)

**Domain of Benefit**	**Metric**	**Result**^a^** (n = 20), No. (%)**
Individual Capacity Building	Improved KT knowledge and skills	18 (90)
	Application of KT knowledge to new projects	13 (65)
	Professional skill development	3 (15)
	Enrolment in PhD	4 (20)
	Job promotion	3 (15)
	New KT/implementation roles	3 (15)
Workforce Capacity Building	Mentor for peers (ad hoc)	14 (70)
	Mentor for peers (formal)	6 (30)
	KT leadership role in team	4 (20)
Enhanced Networks	Part of AH-TRIP network	4 (20)
	External collaborations	5 (25)
Clinical Practice Change	Reduced evidence-practice gap	15 (75)
	Adoption in other services/statewide	5 (25)
Patient/Community Benefits	Improved patient outcomes	5 (25)
Economic Benefits	Reduced healthcare costs	4 (20)
	Investment in model of care	2 (10)
	Reduced implementation waste	3 (15)

Abbreviations: KT, knowledge translation; AH-TRIP, Allied Health Translating Research into Practice. ^a^ Result refers to the impact being evident for each AH-TRIP participant (based on all data available).

####  Individual Capacity Building 

 Increased KT knowledge and skills were reported by almost all AH-TRIP participants. These new skills were subsequently applied to new KT projects, with participants indicating, *“[the AHTRIP program], that’s all I know now… I wouldn’t know what to do if I didn’t use TRIP framework” *(Health Professional [HP] 18). Managers identified that the benefits of participating in AH-TRIP extended to developing other knowledge and skills to support their professional development; for example: *“It’s probably enhanced his [AH-TRIP participant’s] written communication, his understanding of processes and his ability to collaborate with the larger, wider team” *(Manager 3b).New career opportunities were reported by nine AH-TRIP participants, including promotion to more senior roles and new implementation/project roles. Whilst enrolment in a research higher degree was already a career goal for some, one participant said that “*AH-TRIP for me has had a massive impact” *as their “*little QI [quality improvement] project, just got bigger and bigger, applied for my MPhil… and then today I have to submit my PhD upgrade” *(HP 20).

 Participants received recognition for their AH-TRIP projects through presenting at local and national conferences (n = 10, total of 26 presentations) and receiving awards (n = 4, total of 10 awards including four AH-TRIP showcase awards and six awards within their local health service). The importance of “*recognition from senior colleagues, being sought out as an expert in an area with a skill set” *(Manager 1b) and its subsequent impact on staff satisfaction was noted.

####  Workforce Capacity Building 

 Increased capacity for teams/departments to undertake KT as a follow-on effect from the individual participant was reported by participants, with many reporting that they now supported others with KT, mostly through “*pretty informal chats” *with colleagues about “*‘what worked for us,’ ‘what didn’t work for us’ and ‘things that we found tricky’ or ‘why we chose this one [framework] and not this one’” *(HP 17).They also reported identifying when peers may benefit from the AH-TRIP resources or support: “*I kept trying to direct them back to AH-TRIP saying ‘You’ve gotta have a plan’” *(HP 19).

 Some participants reported taking a more active mentorship role including formal project mentoring for peers or taking on a champion role within their department/team to put “*AH-TRIP on the agenda… to create an awareness in the department” *(HP 16).Examples includesetting up local AH-TRIP champion roles and integrating AH-TRIP content into team professional development schedules or meetings. Importantly, managers spoke of the role that AH-TRIP participants have in inspiring their peers: “*It’s a real example to others within our service, as you know what you can do if you have the evidence and if you’ve got the data” *(Manager 3b). Managers also reflected on how they themselves learned about KT from the AH-TRIP participants: “*Because I’ve seen what [HP 9] has done and have learned from her that it’s not quite as simple as just going, ‘Hey, let’s do this’” *(Manager 4b).

 Six participants reported no workforce related impacts from their AH-TRIP involvement. In some cases, this was deemed as not being needed due to existing high levels of capability within their team, or that it was something they planned to do in the future. However, one participant reported attempts to support others in building KT knowledge and skills and involvement in AH-TRIP within their rural site, but these attempts were not successful as* “nobody really got on board with that” *(HP 10).

####  Enhanced Networks 

 Participants reported new or strengthened local networks and new connections with the AH-TRIP team to seek support if needed in future: “*We do know that [AH-TRIP facilitator]…so, if we have any questions, they email straight back. So that’s always good to know as well” *(HP 19). Increased collaboration across disciplines was identified by managers, with the stated benefit of *“raising the profile”* of allied health disciplines “*and what we can do when we all work together” *(Manager 4b). Enhanced external collaborations with new statewide networks, researchers and research institutes, and other health professionals working in other states and internationally were also reported as a result of AH-TRIP which further enhanced individuals’ confidence and ability to undertake KT: *“When she [external researcher] is going to do a project, she’ll come to us and say, ‘Will we get together on this?’ So that kind of networking makes everyone feel a little bit more confident that there’s someone that can help” *(HP 8).

####  Clinical Practice Change 

 Reduction of the evidence-practice gap was achieved in 15 projects completed by AH-TRIP participants. Examples of clinical practice changes included the implementation of multidisciplinary chronic disease management programs, telehealth outreach service, allied health clinical supervision program, fall prevention programs, and allied health delegation models: *“It definitely created change. We went from not having a routine screening tool to implementing a routine so every time a patient came in for treatment, they were getting a screen…And then the outcomes were that we were able to definitely capture more patients at risk” *(HP 15). In three projects, clinical practice change was implemented within a statewide service, and for one project, there was interest in developing a statewide toolkit to support implementation in other health services. The framework and resources developed in one AH-TRIP project were then used to roll-out similar services: *“The work that I’d done around the telehealth model enabled us to really go live and translate a lot of our [other] in-person outpatient services to telehealth very quickly and very efficiently and effectively” *(HP 6).

 In one case, the project resulted in no change to clinical practice. This was reported as a positive outcome because AH-TRIP mentoring helped the participant to realise that practice change was not required: *“We had an extremely low rate of complications, so there really is no problem to address. There was no point in implementing a change in practice because there’s no clinical problem” *(HP 2).In one case, the project could not progress due to contextual barriers related to high workforce turnover and lack of management support. Clinical practice changes could not be assessed for three projects, as they were still underway or paused at the time of evaluation, or outcomes were unknown (due to the participant moving to a new health service).

####  Patient/Community Benefits

 Patient outcomes were only measured and reported in five projects despite 11 projects being reported as completed. Where it was measured, positive patient outcomes were reported, including improvements in nutritional status, diabetes outcomes, access to allied health services and patient experience: *“It does work and it’s well attended and we get good clinical and psychological outcomes from it” *(HP 3).

####  Economic Benefits

 Cost wasonly quantified in one project, where an external health economist calculated cost savings of $AU147 000 for the health service over a 20-month period ($AU88 200 per year): “*522,197 km travel saved [April 2019 to December 2020] = $147,097 PTSS [patient travel subsidy scheme] saved + $74,387 out of pocket expenses saved, average $1,102 per patient” *(HP 6, excerpt from evaluation report). Two projects received recurrent funding from the health service to continue the new model of care as business as usual, representing a financial investment in the sustainment of their project. Economic benefits could be inferred from reported clinical practice change in at least 3 additional projects, where research evidence supports cost savings from falls prevention, multidisciplinary chronic disease management and enhanced malnutrition detection and treatment. Participants also reported that the AH-TRIP initiative itself saves clinician time eg, sharing implementation projects at the Showcase stops “*everyone doing the same mistake over and over again” *(HP 1);AH-TRIPproviding* “a more systematic approach instead of just jumping to a solution… to do something that’s not even addressing what the underlying problem is anyway” *(Manager 2b).

###  Economic Analysis

 An economic analysis could not be undertaken as economic data were only available for one project.

###  Narratives

 The narrative of the AH-TRIP initiative (Table 4) summarises the pathway from program need through to impact and provides contextual understanding to support the interpretation of the modified Payback metrics. In addition, three case studies have been included (Table 5) to illustrate the impact of AH-TRIP for individuals with diverse projects and contexts across metropolitan, regional and rural health services.

**Table 4:** Narrative of the Allied Health Translating Research into Practice Program

**AH-TRIP**	
Background and need	KT is necessary to ensure the provision of evidence-based healthcare, and therefore, it is critical to have a health workforce with the necessary knowledge and skills to undertake the KT Process. A local needs assessment identified that allied health professionals lack knowledge, skills and confidence in implementing research into practice but are interested in engaging in online training and mentoring to develop their skills.
The response	AH-TRIP was developed by research fellows in Queensland Health to build KT capacity amongst the front-line allied health workforce, with a goal of ensuring access to KT expertise and support regardless of the geographical location of the workforce. AH-TRIP consists of (1) training and education (webinars, online resources and case studies), (2) project implementation, (3) support and networks (one-on-one and telementoring, local champions), (4) showcase and recognition, and (5) evaluation. The program is free of charge and participants can choose which aspects of the program they participate in and when.
Key activities	This evaluation identified that participants were generally encouraged to take part in AH-TRIP by a peer, manager or research fellow. They typically found the mentoring component most valuable, and this supported them in using online resources, which they reflected may have been difficult to navigate on their own. Some participants accessed additional external KT training; in some cases, this was before they became involved in AH-TRIP, but sometimes additional training was sought to continue developing their skills. All but two participants completed a KT project as part of their AH-TRIP involvement; one decided that KT was not required as usual care was delivered in line with evidence, and one was not able to progress their project due to contextual barriers.
Impact	Impact of AH-TRIP was evident for individual participants, allied health departments, health services and the community. These include: Improved KT knowledge and skills, now applied to subsequent projects. Research higher degree enrolment, promotions to senior positions and appointment to new implementation roles. Increased informal and formal KT mentoring and leadership in allied health departments. Enhanced KT support networks within AH-TRIP and external groups. Improved delivery of evidence-based practice, with adoption of programs in new sites. Improved patient outcomes. Reduced healthcare costs and implementation waste. Economic impact of AH-TRIP could not be determined due to limitations within the project evaluations (cost data was only collected in one project, and patient outcomes were only collected in five projects).

Abbreviations: KT, knowledge translation; AH-TRIP, Allied Health Translating Research into Practice.

**Table 5:** Narratives of Three Selected Participant Case Studies

**Case 1 (Participant 2: Metropolitan Senior Allied Health Professional)**	
Need	Perceived need to implement a new service to reduce the incidence of postoperative complications.
Progress	At the suggestion of AH-TRIP mentors, baseline data were collected, which demonstrated a low incidence of complications, therefore *“there was no point in implementing a change in practice because there’s no clinical problem.” *The planned project did not proceed.
AH-TRIP support	One-on-one AH-TRIP mentoring (suggested by their manager), then received an AH-TRIP bursary to attend an external 3-day KT course. They then participated in the AH-TRIP telementoring program. They reported using the online resources; however, they reflected that this would not have been enough support without the other AH-TRIP support: *“But if you just sat down with 10 hours to read all the AH-TRIP stuff, it wouldn't you know embed that knowledge.” *They valued the support provided by AH-TRIP “*Really flexible support… when you’re asking for help and that 12 month implementation thing [ telementoring ] was just really well designed and worked.”*
Impact	Individual capacity building: Enhanced KT knowledge: “*you've got to understand what baseline data is, is just step one”*; new implementation-related role *(“It [AH-TRIP] really piqued my interest, and as a result, I worked as the [program implementation] facilitator here for a year”*) which they felt further consolidated their implementation skills. Workforce capacity building: Informal and formal mentoring: shared their AH-TRIP experience with peers and on the AH-TRIP website as a case study for “lessons learned”; now an AH-TRIP champion and viewed as a key resource in the department to support others with TRIP (directing peers to AH-TRIP website and showcase to engage their peers in AH-TRIP). Whilst there is increased knowledge in the workforce, they feel that KT still *“baffles a lot of people.”* Enhanced KT networks: New external collaborations with others undertaking implementation projects.
Factors influencing impact	Enablers: Support from directors and clinical leads *(“It's great to have interested clinicians, but you also need the support of the department and the clinical leads and things to make it doable”*), opportunity to further develop skills within a funded implementation project. Barriers: COVID-19 limited workforce engagement in AH-TRIP *(“COVID has sort of shut down anything non-clinical related... it’s kind of been like a big pause I think”*).
**Case 2 (Participant 20: Senior Allied Health Professional Based in a Regional Area)**	
Need	Post hip-fracture management is not evidence-based.
Progress	This project was still underway, so there were no outcomes to report to date.
AH-TRIP support	Became involved in AH-TRIP after attending a 3-day KT workshop which sparked their interest in AH-TRIP. *“And I was like, ‘ ohh, how can I take this further?’ Then AH-TRIP came along”* They then participated in the AH-TRIP telementoring program: *“the mentors and the team, just the genuine passion and support they provide was, for me personally, really reassuring.”* They also presented at the AH-TRIP showcase. Some project support was also provided by the local research fellow, but acknowledges that this is *“only one person for the whole health service.”*
Domains of benefit	Individual capacity building: Enhanced KT knowledge; now enrolled in a PhD: *“It's put me on a path that I wouldn't think I would be on,”* having met one of their PhD supervisors through AH-TRIP; other professional skill development, with their manager identifying: “*It's probably enhanced his written communication, his understanding of processes and his ability to collaborate with the larger, wider team and also you know it is influencing others within our department as well.”* Workforce capacity building: Formal mentoring of one peer, but they are unsure what influence this has had on the rest of the department. They hope to be a role model for others: *“I think that's, you know, sort of paving the way hopefully for others if they wish.”*
Factors influencing impact	Barriers: Investment of time requires a mind shift of the whole team: *“I think lots of the team are still concerned about the time requirements to actually run a really good QI project where we don't have a lot of fat within our department to make this happen. So you know when they go, everybody's gotta pick up the slack and do their work for them.”*
**Case 3 (Participant 10: Junior Allied Health Professional Based in a Rural Area)**	
Need	Perceived need to improve the goal setting process in inpatient rehabilitation.
Progress	This project had been unable to proceed due to contextual barriers (staff turnover within the implementation team and lack of organisational support).
AH-TRIP support	Participated in AH-TRIP telementoring program (suggested by local research fellow): *“Yeah, they [AH-TRIP people] were really good. Like it's the local support that we don't have.”* Mentoring gave the team a process to follow and support to continue to try to work through their project. *“I think if we hadn’t’ve been enrolled in the AH-TRIP thing, I probably would thrown it in the rubbish bin and walked away because it's just been frustrating the whole time.” *They used some online resources but found the website *“a bit hard to navigate.”*
Domains of benefit	Individual capacity building: Increased knowledge and intention to apply this to change practice. Workforce capacity building: Intention to influence peers’ perspectives and approach to projects; feel confident in sharing their knowledge of KT and AH-TRIP with peers, but there has been limited interest to date *(“nobody really got on board”).*
Factors influencing impact	Barriers: Workforce turnover and retention, meaning that people with skills/experience left and the project team dissolved *(“I was the last man standing”*); lack of management support, with line manager having little knowledge of AH-TRIP except *“the very little bits that I've told her”*; role/permission issues *(“How do you lead a project when you’re not allowed to? Yeah, you’re like being careful of stepping on people’s toes.”)* and poor research culture (“*It sounds terrible that research is not a priority here and realistically, evidence-based stuff probably isn't a priority either. It's more just getting the job done as much as we can*.”)

Abbreviations: KT, knowledge translation; AH-TRIP, Allied Health Translating Research into Practice; QI, quality improvement. Note: Junior professional (Health practitioner level 3); Senior professional (Health practitioner ≥ level 4).

###  Factors influencing the impact of AH-TRIP

**Figure 2:**
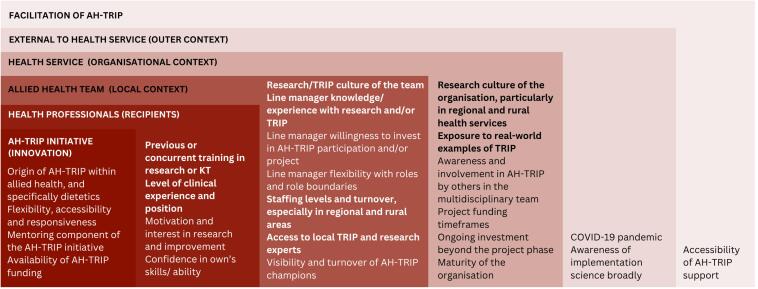
Factors Influencing Impact of the Allied Health-Translating Research into Practice Initiative, Aligned to Constructs of the Integrated Promoting Action of Research Implementation in Health Services Framework.^21^ Abbreviations: KT, knowledge translation; AH-TRIP, Allied Health Translating Research into Practice; ICB, Individual capacity building; WBC, workforce capacity building; EN, enhanced networks; CPC, clinical practice change.

 Barriers and enablers to achieving impact for the workforce and health service were identified across all domains of the i-PARIHS framework and influenced the following Domains of Benefit: Individual Capacity Building, Workforce Capacity Building, Enhanced Networks, and Clinical Practice Change (Figure 2). Most factors manifested as both barriers and enablers, depending on the local setting and recipients within that setting (Supplementary file 3, Table S2).

## Discussion

 This evaluation has described the impact of a KT capacity building program on allied health professionals, health services, and systems, through applying the FAIT framework. Additionally, barriers and enablers to the program achieving impact were also explored. Results indicated that health service investment in a multimodal KT capacity building program can have broad reaching, multi-level impacts. In our work we observed this at the level of the individual (KT capacity building, professional skill development, career progression, enhanced networks), workforce (informal and formal KT mentoring and leadership) and health service (reduced evidence-practice gap, improved patient care and outcomes, new models of care funded, cost savings and reduced implementation waste).

 With an overwhelming number of KT practice tools available in the literature,^27^ the AH-TRIP curation of content delivered alongside mentorship to assist health practitioners in choosing and applying KT frameworks and theories supported individuals to build skills and knowledge in implementation practice. Developing competencies in collaboration knowledge and skills through networking and building implementation teams across disciplines has been identified as a critical factor in the success of other KT capacity building programs.^28^ The development and growth of AH-TRIP within the allied health discipline was seen as a strength for internal engagement of allied health professionals. However, this limited the impact of KT projects and networks where there was a poor understanding of KT in other disciplines (eg, nursing and medicine) who often support or make key decisions about implementation of evidence into practice. Interdependence with other disciplines and professionals has been identified as a key feature of the allied health practice context for KT,^29^ and therefore, designing and implementing KT capacity building programs for multidisciplinary teams rather than within specific discipline groups should be considered.

 Allied health practitioners are known to be intrinsically motivated to participate in research and remain on the “cutting edge,” but organisational factors are burdensome.^30^ Organisational barriers to utilising research findings in practice are not new nor unique to allied health disciplines. For the last 20 years, nurses have reported a range of organisational factors limiting KT in practice including high workloads, insufficient time and resources for research, lack of cooperation and support from the administration and/or clinical team, workplace culture resistance to change and lack of authority to change patient care.^31,32^ The local context in which AH-TRIP participants conducted their KT activity was highly variable. Contextual barriers, for example research culture, access to research capable line managers or research fellows, staff turnover etc, significantly influenced impact of the AH-TRIP program for participants. This was more pronounced in regional and rural health services. Challenges of building workforce capacity in regional, rural and remote areas are not unique to this evaluation, with other studies reporting low levels of research funding, chronic workforce shortages and tension between undertaking research and delivering clinical care.^33-35^ This suggests the need to invest in understanding the unique regional, rural and remote contextual barriers to KT in order to adapt existing KT capacity building programs and/or develop targeted implementation strategies to support them.

 The organisational barriers identified may also have been influenced by the fact that there was no program-facilitated mechanism for health practitioners to connect directly with health service policy-makers. Integrated KT^36^ is a strategy that holds great promise to improve KT and is defined by researchers and decision-makers engaging together in mutually beneficial projects.^37^ Integrated KT strategies that embed policy-makers within interdisciplinary project teams could strengthen some of the gaps in the domains of benefit seen in this evaluation, such as lack of measures for return on investment or economic assessments. Reducing the evidence-practice gap was achieved in almost all projects; however, quantifiable measures of patient outcomes, economic benefit and changes to policy and legislation were rarely available. AH-TRIP was designed for KT capacity building of front-line clinical workers and most of its impacts were achieved through a “bottom-up” engagement of clinicians with the program. This meant that measures of project success, perhaps appropriately, tended to focus on changes in clinical processes and practices proven by research to create patient and/or economic benefits. To further support planning of and understanding patient benefits consumer engagement and partnerships will be valuable. There is increasing interest and unrealised potential in including health consumers (ie, current, past or potential users of health services) as integrated KT teams.^38^ Building skills in consumer engagement and partnerships within KT capacity building programs may support the inclusion of patient outcome and experience measures within KT project evaluation plans. This would provide evidence for the benefits for consumers and the community, which was lacking in projects included in the current evaluation. Recent competencies for integrated KT^39^ could be considered when updating the AH-TRIP initiative to facilitate the inclusion of consumers and policy-makers in future KT projects.

 This impact evaluation reinforces that key factors for success remain unchanged since the review of Slade et al over five years ago. In this review it was indicated that sustainability of change requires (*a*) people and structures that value evidence-based practice, (*b*) research capability and literacy of healthcare leaders and managers, (*c*) an organisational research culture, and (*d*) a motivated workforce with skills and capability.^40^ There are few KT interventions that have evaluated long term sustainability.^14,41^ Significant organisational barriers to KT capacity building through AH-TRIP, as identified in the results, were the short timeframes of projects and lack of ongoing funding for successful innovations which seems to be a common barrier in healthcare services.^42,43^ This highlights the need to accompany initiatives like AH-TRIP with initiatives aimed at health service leaders to create the necessary conditions for individuals skilled in KT to apply these skills to improve health service delivery at the front-line. Integration of these programs across the health system at all levels may support the impact by increasing middle managers’ knowledge of and experience with KT, identified as an enabler in this study and others.^14^

 A strength of this evaluation was the use of a specific research impact evaluation framework to guide data analysis. To our knowledge, this is the first time that FAIT has been applied to KT capacity building programs. Our experience suggests that there are some similar domains of benefit to research impact, but there are some domains of benefit unique to KT and capacity building focused initiatives. Applying FAIT to understand KT capacity building programs allows a richer and deeper understanding of problems in a multi-dimensional way to overcome limitations of existing tools and frameworks.

 A further strength is the consideration of impact alongside context, acknowledging that some individuals or projects might succeed due to contextual factors related to the culture, resources or networks within their team or organisation.^44^ Limitations of this evaluation include our sampling method, which may have introduced self-selection bias through participants being more likely to volunteer where there had been a positive impact from AH-TRIP. Response bias may have led to participants overstating the impact of AH-TRIP in an attempt to please the researchers; however, the use of an interviewer independent from AH-TRIP, ensuring anonymity of responses and asking participants to provide evaluation reports/papers to substantiate the impact were strategies used to reduce this bias. As a multicomponent intervention, it is not possible to determine whether participating in specific components of the AH-TRIP initiative resulted more or less impact. This could have been explored if we had invited participants who engaged only with the education and training aspects of AH-TRIP; however, as the online platform does not collect data on individual users, it was impossible to identify these people. Despite attempts to demonstrate the impact in domains such as clinical practice change, consumer and economic benefits, empirical data were generally lacking. Applying an impact evaluation framework prospectively when supporting AH-TRIP project planning and reporting may improve the quality of data in the future. Conceptualisation of impact as “domains of benefits” within the FAIT framework may have limited exploration of potential unintended negative impacts of the AH-TRIP program in this evaluation^44^; this could be considered in future evaluation of the program and in the refinement of research impact frameworks such as FAIT.

## Conclusions

 This impact evaluation identified that both allied health professionals and the broader health service and system benefit in many ways from a training and support program designed to enhance their KT practices within dynamic environments. Prospective planning for impact may improve the availability of empirical data in future evaluations. The impact from such initiatives may be enhanced by addressing contextual barriers related to workforce, research capacity and funding models. Continued delivery of the program to develop KT workforce across the health workforce may support this change by skilling current front-line workers who may become future health service leaders who are experienced in KT, able to champion its value, and are prepared and able to influence organisational barriers to enable greater impact.

## Acknowledgements

 The authors thank all those who have participated in or supported AH-TRIP, in particular those individuals who participated in this evaluation.

## Disclosure of artificial intelligence (AI) use

 Not applicable.

## Ethical issues

 This project was approved as a program evaluation by the hospital human research ethics committee (LNR/2019/QRBW/57225). All participants provided written or verbal consent to participate. All identifiable data was stored on the university OneDrive with access for authorised users only. All data were then de-identified (names removed of individuals and health services) prior to sharing amongst the research team.

## Conflicts of interest

 Rachelle Pitt is employed by the Queensland Health department that funds AH-TRIP and contributed funding for this evaluation. Ashley Cameron is employed as the AH-TRIP Statewide Program Manager; Nina Meloncelli is employed as the AH-TRIP Workforce Development Office, Metro North Health. All authors (with the exception of Alita Rushton) are members of the AH-TRIP Evaluation Working Group.

## Supplementary files


Supplementary file 1 contains Table S1.

Supplementary file 2. Interview Guide and Pre-interview Survey.

Supplementary file 3 contains Table S2.

## References

[1] Sarkies MN, Francis-Auton E, Long JC, Pomare C, Hardwick R, Braithwaite J (2022). Making implementation science more real. BMC Med Res Methodol.

[2] Grimshaw JM, Eccles MP, Lavis JN, Hill SJ, Squires JE (2012). Knowledge translation of research findings. Implement Sci.

[3] Graham ID, Logan J, Harrison MB (2006). Lost in knowledge translation: time for a map?. J Contin Educ Health Prof.

[4] Glasziou P, Altman DG, Bossuyt P (2014). Reducing waste from incomplete or unusable reports of biomedical research. Lancet.

[5] King OA, Sayner A, Beauchamp A, Hitch D, Aras D, Wong Shee A (2023). Translating research into rural health practice: a qualitative study of perceived capability-building needs. Rural Remote Health.

[6] Reszel J, Daub O, Leese J (2023). Essential content for teaching implementation practice in healthcare: a mixed-methods study of teams offering capacity-building initiatives. Implement Sci Commun.

[7] Moore JE, Rashid S, Park JS, Khan S, Straus SE (2018). Longitudinal evaluation of a course to build core competencies in implementation practice. Implement Sci.

[8] Park JS, Moore JE, Sayal R (2018). Evaluation of the “Foundations in Knowledge Translation” training initiative: preparing end users to practice KT. Implement Sci.

[9] Barrimore SE, Cameron AE, Young AM, Hickman IJ, Campbell KL (2020). Translating research into practice: how confident are allied health clinicians?. J Allied Health.

[10] Young AM, Olenski S, Wilkinson SA (2020). Knowledge translation in dietetics: a survey of dietitians’ awareness and confidence. Can J Diet Pract Res.

[11] Harris C, Allen K, Waller C (2017). Sustainability in Health care by Allocating Resources Effectively (SHARE) 7: supporting staff in evidence-based decision-making, implementation and evaluation in a local healthcare setting. BMC Health Serv Res.

[12] Young AM, Cameron A, Meloncelli N (2023). Developing a knowledge translation program for health practitioners: Allied Health Translating Research into Practice. Front Health Serv.

[13] Hickman IJ, Cameron AE, McRae P (2021). Feasibility and acceptability of a pilot knowledge translation telementoring program for allied health professionals. Int J Allied Health Sci Pract.

[14] King O, West E, Alston L (2024). Models and approaches for building knowledge translation capacity and capability in health services: a scoping review. Implement Sci.

[15] Deeming S, Searles A, Reeves P, Nilsson M (2017). Measuring research impact in Australia’s medical research institutes: a scoping literature review of the objectives for and an assessment of the capabilities of research impact assessment frameworks. Health Res Policy Syst.

[16] Raftery J, Hanney S, Greenhalgh T, Glover M, Blatch-Jones A (2016). Models and applications for measuring the impact of health research: update of a systematic review for the Health Technology Assessment programme. Health Technol Assess.

[17] Cruz Rivera S, Kyte DG, Aiyegbusi OL, Keeley TJ, Calvert MJ (2017). Assessing the impact of healthcare research: a systematic review of methodological frameworks. PLoS Med.

[18] Searles A, Doran C, Attia J (2016). An approach to measuring and encouraging research translation and research impact. Health Res Policy Syst.

[19] Dodd R, Ramanathan S, Angell B (2019). Strengthening and measuring research impact in global health: lessons from applying the FAIT framework. Health Res Policy Syst.

[20] Paul CL, Verrills NM, Ackland S (2024). The impact of a regionally based translational cancer research collaborative in Australia using the FAIT methodology. BMC Health Serv Res.

[21] Harvey G, Kitson A (2016). PARIHS revisited: from heuristic to integrated framework for the successful implementation of knowledge into practice. Implement Sci.

[22] Tovin MM, Wormley ME (2023). Systematic development of standards for mixed methods reporting in rehabilitation health sciences research. Phys Ther.

[23] Queensland Government. Queensland Population Projections 2002-2026. 2021. Available from: https://public.tableau.com/app/profile/tim.roselli/viz/HHSpopulationprojections/Display. Accessed January 22, 2024.

[24] Malterud K, Siersma VD, Guassora AD (2016). Sample size in qualitative interview studies: guided by information power. Qual Health Res.

[25] St Louis Bernard Becker Medical Library. The Becker List: Impact Indicators. 2014. Available from: https://becker.wustl.edu/impact-assessment/files/becker_model-reference.pdf. Accessed September 5, 2022.

[26] Elo S, Kyngäs H (2008). The qualitative content analysis process. J Adv Nurs.

[27] Bhuiya AR, Sutherland J, Boateng R (2024). A scoping review reveals candidate quality indicators of knowledge translation and implementation science practice tools. J Clin Epidemiol.

[28] Schultes MT, Aijaz M, Klug J, Fixsen DL (2021). Competences for implementation science: what trainees need to learn and where they learn it. Adv Health Sci Educ Theory Pract.

[29] Hitch D, Pepin G, Lhuede K, Rowan S, Giles S (2019). Development of the translating allied health knowledge (TAHK) framework. Int J Health Policy Manag.

[30] Pager S, Holden L, Golenko X (2012). Motivators, enablers, and barriers to building allied health research capacity. J MultidiscipHealthc.

[31] Berthelsen C, Hølge-Hazelton B (2021). The importance of context and organization culture in the understanding of nurses’ barriers against research utilization: a systematic review. Worldviews Evid Based Nurs.

[32] Williams B, Perillo S, Brown T (2015). What are the factors of organisational culture in health care settings that act as barriers to the implementation of evidence-based practice? A scoping review. Nurse Educ Today.

[33] Wong Shee A, Quilliam C, Corboy D (2022). What shapes research and research capacity building in rural health services? Context matters. Aust J Rural Health.

[34] Wakerman J, Humphreys J, Russell D (2019). Remote health workforce turnover and retention: what are the policy and practice priorities?. Hum Resour Health.

[35] Battye K, Roufeil L, Edwards M, Hardaker L, Janssen T, Wilkins R. Strategies for increasing allied health recruitment and retention in Australia: A Rapid Review. Services for Australian Rural and Remote Allied Health (SARRAH); 2019.

[36] Kothari A, Wathen CN (2013). A critical second look at integrated knowledge translation. Health Policy.

[37] Gagliardi AR, Berta W, Kothari A, Boyko J, Urquhart R (2016). Integrated knowledge translation (IKT) in health care: a scoping review. Implement Sci.

[38] Banner D, Bains M, Carroll S (2019). Patient and public engagement in integrated knowledge translation research: are we there yet?. Res InvolvEngagem.

[39] Yeung E, Scodras S, Salbach NM, Kothari A, Graham ID (2021). Identifying competencies for integrated knowledge translation: a Delphi study. BMC Health Serv Res.

[40] Slade SC, Philip K, Morris ME (2018). Frameworks for embedding a research culture in allied health practice: a rapid review. Health Res Policy Syst.

[41] Tricco AC, Ashoor HM, Cardoso R (2016). Sustainability of knowledge translation interventions in healthcare decision-making: a scoping review. Implement Sci.

[42] Tieosapjaroen W, Chen E, Ritchwood T (2024). Designathons in health research: a global systematic review. BMJ Glob Health.

[43] Greenhalgh T, Papoutsi C (2019). Spreading and scaling up innovation and improvement. BMJ.

[44] Reed MS, Rudman H (2023). Re-thinking research impact: voice, context and power at the interface of science, policy and practice. Sustain Sci.

