# Supplementary material for: Mixed Methods Evaluation of the Impact of Allied Health – Translating Research into Practice (AH-TRIP) Program on the Knowledge Translation Capacity of the Allied Health Workforce
Source: Int J Health Policy Manag. 2025 Oct 27;14:8910. doi: 10.34172/ijhpm.8910 (PMC12958154; doi:10.34172/ijhpm.8910)
Supplement: Supplementary file 1 — contains Table S1. [file ijhpm-14-8910-s001.pdf]

Article title: Mixed Methods Evaluation of the Impact of Allied Health – Translating Research into Practice (AH-TRIP) Program on the Knowledge Translation Capacity of the Allied Health Workforce

Journal name: International Journal of Health Policy and Management (IJHPM)

Authors' information: Adrienne M. Young[1,2]*, Alita Rushton[1,3], Ashley Cameron[4,5], Nina Meloncelli[6,5], Shelley A. Wilkinson[7,8], Rachelle Pitt[4], Kathryn McFarlane[9], Katrina L. Campbell[1,5], Gillian Harvey[10], Ingrid J. Hickman[11]

[1]Centre for Health Services Research, Faculty of Medicine, The University of Queensland, Brisbane, QLD, Australia.

[2]Dietetics and Food Services, Royal Brisbane and Women's Hospital, Brisbane, QLD, Australia.

[3]School of Health Sciences and Social Work, Griffith University, Gold Coast, QLD, Australia.

[4]Office of the Chief Allied Health Officer, Queensland Health, Brisbane, QLD, Australia.

[5]Healthcare Excellence and Innovation, Metro North Hospital and Health Service, Brisbane, QLD, Australia.

[6]Office of the Chief Allied Health Practitioner, Metro North Allied Health, Brisbane, QLD, Australia.

[7]Department of Obstetric Medicine, Mater Mothers' Hospitals, Brisbane, QLD, Australia.

[8]School of Pharmacy, Faculty of Health and Behavioural Sciences, The University of Queensland, Brisbane, QLD, Australia.

[9]Allied Health, Cairns and Hinterland Hospital and Health Service, Cairns, QLD, Australia.

[10]Caring Futures Institute, College of Nursing and Health Sciences, Flinders University, Adelaide, SA, Australia.

[11]ULTRA Team, Clinical Trial Capability, Centre for Clinical Research, The University of Queensland, Brisbane, QLD, Australia.

*Correspondence to: Adrienne M. Young; Email: a.m.young@uq.edu.au

Citation: Young AM, Rushton A, Cameron A, et al. Mixed methods evaluation of the impact of Allied Health – Translating Research into Practice (AH-TRIP) program on the knowledge translation capacity of the allied health workforce. Int J Health Policy Manag. 2025;14:8910. doi:10.34172/ijhpm.8910

Supplementary file 1

Table S1. Mixed Methods Reporting in Rehabilitation & Health Sciences Checklist

Instruction	Y/N; comments
**TITLE**	
Concisely describes the topic of the study identifying the study as mixed methods	Y
**INTRODUCTION/ BACKGROUND**	
Summarizes key elements using *journal specific* abstract format; For example: Introduction, Methods, Results, Discussion, and Significance/potential impact to rehabilitation and/or societal health	Y
Includes literature review on the topic of interest (quantitative, qualitative, and mixed)	Y
Identifies gap that justifies the need for mixed methods approach	Y – page 3, lines 28-31
Clearly states overarching goal of the study that supports a mixed methods approach	Y- page 5, lines 1-4
States the rationale for using mixed methods research	Y – page 3, lines 28-31
Clearly identifies discrete aim(s) for qualitative and quantitative components	N/A – single aim however the framework used necessitates mixed methods (as explained in the methods)
Aims align with corresponding component methods	N/A
Provides statement of significance and potential impact	Y – page 3, lines 30-31
**METHODS**	
**Design –** Clearly describes the mixed methods design (exploratory sequential, explanatory sequential, concurrent, etc.) used to accomplish the overarching goal of the project:<br>• *Emphasis noted* (i.e., Sequential QUAL--> quan or QUAN--> qual; Concurrent QUAL + QUAN)<br>• *Visual display of overall design highlighting integration* (e.g., model, flow chart, figure)	Y – page 5, lines 14-15
Describes and supports the qualitative and quantitative methodologies (phenomenology, randomized control trial) used to accomplish the discrete aim(s) of the project	Y – page 5, lines 7-21

States researcher(s) background and contributions to project (e.g. content or methods expertise, relationships to participants)	Y – page 8, lines 10-12
Identifies setting (e.g. hospital system, geographical location)	Y – page 7, lines 2-3
**Subjects/Participants** - Clearly describes and supports the following:<br>• Sampling and recruitment<br>• Inclusion/Exclusion criteria<br>• Ethical considerations (consent process, researcher relationship with participants)	Y<br><br>Page 7, lines 14-29.<br><br>Page 9, lines 27-28
**Data collection -** Clearly describes and supports the following:<br>• Pilot study (if applicable)<br>• Instrumentation (validity, reliability)<br>• Implementation matrix (e.g. data source, timeline, type, anticipated outcomes)	Y - page 8, lines 6-8<br>N/A
**Data analysis -** Clearly states and describes analysis procedures for:<br>• Qualitative<br>• Quantitative<br>• Mixed Methods (integration)	Y – page 8, lines 31-33<br>Y – page 8, lines 29-31<br>Y – page 8, line 26
**Methodological Rigor** – Clearly describes steps taken to establish rigor:<br>• Qualitative (e.g. credibility, dependability, confirmability, transferability)<br>• Quantitative (e.g. validity, reliability, generalizability)<br>• Mixed Methods (validity or legitimacy)	Y – page 8, line 33; page 9, lines 1-3, 13-16.
RESULTS/FINDINGS	
**Clearly presents findings for study components:**<br>• Qualitative (includes data exemplars)<br>• Quantitative<br>• Mixed Methods: Provides integrated findings/overall study results (e.g., joint display)	Y – pages 12-16; page 20<br>Y - page 10, lines 6-14;<br>Y – page 7-19
**DISCUSSION**	

• *Incorporates discussion on implications of integrated findings*<br>• Provides synthesis and interpretation of findings in the context of existing literature and theoretical/conceptual framework<br>• Includes subsection of limitations	Y – pages 22-25<br><br>Y – page 25

Reference: Tovin MM, Wormley ME. Systematic Development of Standards for Mixed Methods Reporting in Rehabilitation Health Sciences Research. Phys Ther. 2023 Nov 4;103(11):pzad084. doi: 10.1093/ptj/pzad084. PMID: 37672215.
