# Supplementary material for: Mixed Methods Evaluation of the Impact of Allied Health – Translating Research into Practice (AH-TRIP) Program on the Knowledge Translation Capacity of the Allied Health Workforce
Source: Int J Health Policy Manag. 2025 Oct 27;14:8910. doi: 10.34172/ijhpm.8910 (PMC12958154; doi:10.34172/ijhpm.8910)
Supplement: Supplementary file 2 — Interview Guide and Pre-interview Survey. [file ijhpm-14-8910-s002.pdf]

Article title: Mixed Methods Evaluation of the Impact of Allied Health – Translating Research into Practice (AH-TRIP) Program on the Knowledge Translation Capacity of the Allied Health Workforce

Journal name: International Journal of Health Policy and Management (IJHPM)

Authors' information: Adrienne M. Young[1,2]*, Alita Rushton[1,3], Ashley Cameron[4,5], Nina Meloncelli[6,5], Shelley A. Wilkinson[7,8], Rachelle Pitt[4], Kathryn McFarlane[9], Katrina L. Campbell[1,5], Gillian Harvey[10], Ingrid J. Hickman[11]

[1]Centre for Health Services Research, Faculty of Medicine, The University of Queensland, Brisbane, QLD, Australia.

[2]Dietetics and Food Services, Royal Brisbane and Women's Hospital, Brisbane, QLD, Australia.

[3]School of Health Sciences and Social Work, Griffith University, Gold Coast, QLD, Australia.

[4]Office of the Chief Allied Health Officer, Queensland Health, Brisbane, QLD, Australia.

[5]Healthcare Excellence and Innovation, Metro North Hospital and Health Service, Brisbane, QLD, Australia.

[6]Office of the Chief Allied Health Practitioner, Metro North Allied Health, Brisbane, QLD, Australia.

[7]Department of Obstetric Medicine, Mater Mothers' Hospitals, Brisbane, QLD, Australia.

[8]School of Pharmacy, Faculty of Health and Behavioural Sciences, The University of Queensland, Brisbane, QLD, Australia.

[9]Allied Health, Cairns and Hinterland Hospital and Health Service, Cairns, QLD, Australia.

[10]Caring Futures Institute, College of Nursing and Health Sciences, Flinders University, Adelaide, SA, Australia.

[11]ULTRA Team, Clinical Trial Capability, Centre for Clinical Research, The University of Queensland, Brisbane, QLD, Australia.

*Correspondence to: Adrienne M. Young; Email: a.m.young@uq.edu.au

Citation: Young AM, Rushton A, Cameron A, et al. Mixed methods evaluation of the impact of Allied Health – Translating Research into Practice (AH-TRIP) program on the knowledge translation capacity of the allied health workforce. Int J Health Policy Manag. 2025;14:8910. doi:10.34172/ijhpm.8910

Supplementary file 2. Interview Guide and Pre-interview Survey

Interview guide

1. **Can you tell me about the impacts of your AH-TRIP project? (project level –** *wording modified to suit the specific project***)**

Prompts:

- How has the project impacted patient care or outcomes? How has it changed clinical practice? (i.e. delivery of healthcare in line with evidence)
- How has the project impacted on outcomes for staff? (e.g. staff satisfaction, workloads)
- Has there been an impact more broadly for your health service as a result of your project? (e.g. changes to policies/procedures/guidelines; implementation elsewhere)
- Do you know of any cost/efficiency outcomes for the health service as a result of your project?
    - Do they feel that the time/cost invested in the project was outweighed or greater than the benefit associated with the TRIP project?

Ask in pre-interview survey – clarify any further details in interview

- Have you had any publications from your AH-TRIP project?
- Has your AH-TRIP project received any awards or recognition?
- Have you presented at any conferences on your AH-TRIP project?
- Was your work disseminated anywhere else? (network meetings, local presentations, newsletters/broadcasts, social media, etc)
- Did you receive funding or FTE/time to undertake your AH-TRIP project? What was the cost of implementing the project? Are there any ongoing costs required for delivery/sustainability?

2. **How has AH-TRIP contributed to your personal and professional development? (individual level)**

Prompts:

- How was it impacted on your knowledge?
    - How do you feel your knowledge regarding TRIP has changed since participating in AH-TRIP?
    - Has this affected your understanding of TRIP more broadly?
    - Has your approach to research and quality projects changed since engaging with TRIP?
    - Have you discovered or developed any new knowledge as a result of your participation in AH-TRIP?

  - Have you had any influence on policy or guideline changes?

- Has there been any 'flow on' effects for you related to your participation in AH-TRIP?
  - Have you taken on any new research endeavours?
  - Have you commenced (or considered) a higher degree by research?
  - Have you taken on any new roles or career opportunities as a result of your engagement with AH-TRIP?
  - Have you personally received any awards or recognition as a result of your engagement with AH-TRIP?
  - Did participating in AH-TRIP affect your collaborations/networks/partnerships?
- Did participating in AH-TRIP cost you anything? (time: work, own; PD fees)
  - Was the cost/time outweighed or greater than the benefit associated with your engagement/participation in AH-TRIP?

3. **How has your participation in AH-TRIP changed how things are done in your team, department or hospital more broadly? (team/department level)**

Prompts:

- Have there been any 'flow on' effects for the research culture, engagement, broader partnerships or collaborations (MDT, other HHS, etc)
- Have there been changes to the processes/operations related to AH-TRIP? E.g.:
  - orientation guidelines, local instructions, etc
  - guidelines or policies regarding AH-TRIP?
  - KPIs in your team regarding AH-TRIP? (e.g. # of TRIP projects, interaction with AH-TRIP online webinars, showcase, mentoring)
- Who has been leading/ championing these changes? What has your role been?
- Are there any mandatory roles/positions/representation of AH-TRIP within your team/department?
  - Do you undertake any of these roles?
- Do you know if any of this work has added any costs to the department? (PD fees, FTE cost, etc)
  - Were these costs outweighed or greater than the benefit associated with your engagement/ participation in AH-TRIP?

4. **Has your participation in AH-TRIP had any flow-on effects for other staff in your department/ward?**

Prompts:

- Has anyone else undertaken AH-TRIP projects?

    - Were there any changes to practice from these?
    - Do you know of any cost/efficiency outcomes for the health service from other projects undertaken in your department (related to your involvement in TRIP)?
- How do you feel the knowledge of TRIP amongst those in your broader team/department has changed since your participation in AH-TRIP?
- Has staff satisfaction changed related to involvement in AH-TRIP broadly?
