# Supplementary material for: Mixed Methods Evaluation of the Impact of Allied Health – Translating Research into Practice (AH-TRIP) Program on the Knowledge Translation Capacity of the Allied Health Workforce
Source: Int J Health Policy Manag. 2025 Oct 27;14:8910. doi: 10.34172/ijhpm.8910 (PMC12958154; doi:10.34172/ijhpm.8910)
Supplement: Supplementary file 3 — contains Table S2. [file ijhpm-14-8910-s003.pdf]

Article title: Mixed Methods Evaluation of the Impact of Allied Health – Translating Research into Practice (AH-TRIP) Program on the Knowledge Translation Capacity of the Allied Health Workforce

Journal name: International Journal of Health Policy and Management (IJHPM)

Authors' information: Adrienne M. Young[1,2]*, Alita Rushton[1,3], Ashley Cameron[4,5], Nina Meloncelli[6,5], Shelley A. Wilkinson[7,8], Rachelle Pitt[4], Kathryn McFarlane[9], Katrina L. Campbell[1,5], Gillian Harvey[10], Ingrid J. Hickman[11]

[1]Centre for Health Services Research, Faculty of Medicine, The University of Queensland, Brisbane, QLD, Australia.

[2]Dietetics and Food Services, Royal Brisbane and Women's Hospital, Brisbane, QLD, Australia.

[3]School of Health Sciences and Social Work, Griffith University, Gold Coast, QLD, Australia.

[4]Office of the Chief Allied Health Officer, Queensland Health, Brisbane, QLD, Australia.

[5]Healthcare Excellence and Innovation, Metro North Hospital and Health Service, Brisbane, QLD, Australia.

[6]Office of the Chief Allied Health Practitioner, Metro North Allied Health, Brisbane, QLD, Australia.

[7]Department of Obstetric Medicine, Mater Mothers' Hospitals, Brisbane, QLD, Australia.

[8]School of Pharmacy, Faculty of Health and Behavioural Sciences, The University of Queensland, Brisbane, QLD, Australia.

[9]Allied Health, Cairns and Hinterland Hospital and Health Service, Cairns, QLD, Australia.

[10]Caring Futures Institute, College of Nursing and Health Sciences, Flinders University, Adelaide, SA, Australia.

[11]ULTRA Team, Clinical Trial Capability, Centre for Clinical Research, The University of Queensland, Brisbane, QLD, Australia.

*Correspondence to: Adrienne M. Young; Email: a.m.young@uq.edu.au

Citation: Young AM, Rushton A, Cameron A, et al. Mixed methods evaluation of the impact of Allied Health – Translating Research into Practice (AH-TRIP) program on the knowledge translation capacity of the allied health workforce. Int J Health Policy Manag. 2025;14:8910. doi:10.34172/ijhpm.8910

Supplementary file 3

**Table S2.** Factors influencing impact of the Allied Health-Translating Research into Practice (AH-TRIP) initiative, aligned to constructs of the integrated Promoting Action of Research Implementation in Health Services (i-PARIHS) Framework			
**i-PARIHS construct**	**Factor**	**Barrier and/or enabler**	**Exemplar quote**
Facilitation (enabling implementation of the innovation)	Accessibility of AH-TRIP support	Enabler	*"I do feel like the staff [AH-TRIP team] were like really like just really helpful and approachable and friendly, so I would e-mail them if I sort of had an outstanding, you know, if I wanted to get back in, or you know what I mean, like sort of, yeah, access that again." HP 14*
AH-TRIP initiative itself (the innovation)	Origin of AH-TRIP within allied health (and specifically dietetics)	Enabler (dietetics) Barrier (other disciplines)	*"We were the first site to implement AH-TRIP, so we have the person that designed it and led it. So I think for us, definitely, we're probably ahead of most areas of understanding it, and its use, and having people that would assist us with implementing it." HP 4*<br>*"It's been more cultural kind of awareness of the importance of implementation science and with AH-TRIP being mentioned all the time, you know, so it's coming up within the Dietetic framework all the time. Dietitians are amazing, lead the way all the time, don't they?!" HP 8*<br>*"I don't know whether there's a perception that AH-TRIP is very dietetics focused, and not in a bad way, but I don't know whether that means that there's an understanding from other disciplines that they can actually access the support from AH-TRIP." HP 7*<br>*"I understand there's some focus on moving it away from Allied Health to TRIP across all streams. And I think that's really important. I know often allied health have led the way in these sorts of innovations, but I think it'll be good for it to become not just allied health focus so that our medical and nursing colleagues [become involved]. HP 6*
**i-PARIHS construct**	**Factor**	**Barrier and/or enabler**	**Exemplar quote**

AH-TRIP initiative itself (the innovation) cont…	Flexibility, accessibility and responsiveness of the initiative	Enabler	*“Really flexible support… when you’re asking for help and that 12 month [telementoring]was just really well designed and worked” HP 2*
	Mentoring component of the AH-TRIP initiative	Enabler	*“Yeah, they [AH-TRIP mentoring panel] were really good. Like it's the local support that we don't have. I think if we hadn’t been enrolled in the AH-TRIP thing, I probably would have thrown it in the rubbish bin and walked away because it's just been frustrating the whole time.” HP 10*<br>*“It was good to be able to meet with someone else who's already gone through projects like this. You know, going ‘ohh, yeah, they do that sometimes. You just gotta let them go.’” HP 9*
	Availability of AH-TRIP funding	Enabler	*“You need more [time/funding] than what you can do in your current workload to do it, but to access smaller pockets of money and funding can actually be really productive and create really big change as well.” Manager 1b*
AH-TRIP participants (the recipient)	Previous or concurrent training in research or KT	Enabler	*“I was fortunate enough to be able to go to the [research centre] three-day knowledge translation course as well, and that had a profound impact on my understanding” HP 1*<br>*“The whole AH-TRIP initiative in some ways came after being introduced to implementation science and models and frameworks, so my taking on the AH-TRIP champion role was probably a product of me learning about these concepts through the [university] Grad Cert program.” HP 6*
	Level of clinical experience and position	Barrier (junior staff)<br>Enabler (team leader/ managers)	*“I'm a HP3. I'm like supposedly a junior person not a senior person. There's been no senior support at all. There's been no like management support either. So we're kind of still stuck and trapped.” HP 10*<br>*“As the new director here, I guess I'm as a minimum expecting that everybody would be involved in, if not leading some kind of QI.” HP 6*
**i-PARIHS construct**	**Factor**	**Barrier and/or enabler**	**Exemplar quote**

AH-TRIP participants (the recipient) cont…	Motivation and interest in research and improvement	Barrier<br>Enabler	*"So I think that it is very much there, but it's probably not on everyone's front of mind, but I think that's just the dynamics of a department that has a varying level of experience and skill and where they're at in their career and what their interests are." HP 15*<br>*"It was like part of staff members' goals to be able to present something to the AH-TRIP showcase" HP 1*<br>*"I think just naturally, my own slant on research is more translational style of research where you want to see an actual outcome for the patients." HP 16*
	Confidence in own's skills/ ability	Barrier	*[When asked if they'd consider being an AH-TRIP champion]: "Aw probably, I mean, I don't know if I'm, yeah, in the future potentially, when I feel more comfortable with it." HP 13*
Allied health department/ team (the local context)	Research/TRIP culture of the department/team	Barrier<br>Enabler	*"I definitely feel there should be [more impact]. But I'm one person. So how much can I influence?" HP 20*<br>*"There's been a few in-services and a couple of other projects done that have just put it on the radar. And it's probably just trickling in amongst all the other research things that are going on. So it's one piece of other research approaches that that are going on." HP 16*<br>*"Unless it's kind of put into our structured education time, it relies on a very motivated individual to sort of seek it out. There isn't always the time for that." HP 11*<br>*"We want to make sure that we're doing the best things for the best reasons in the best way. And I think this departments excellent at that." HP 3*
	Line manager with research and/or TRIP knowledge and/or experience	Enabler<br>Barrier	*"I've got a PhD so I've done quite a bit of research and then was very interested when AH-TRIP sort of first started coming around" Manager 5b*<br>*"[My manager only knows] the very little bits [about AH-TRIP] that I've told her" HP 10*
**i-PARIHS construct**	**Factor**	**Barrier and/or enabler**	**Exemplar quote**

Allied health department/ team (the local context) cont…	Support from line managers<br>- Willingness to invest in participants to undertake AH-TRIP projects and professional development<br>- Flexibility with role boundaries	Enabler<br>Barrier	*“Yes they'll [the department] give you a set number of days that we then apply to our project to utilise as we see fit basically. Each team is given this set allocation of days” HP 11*<br>*“But you know when [HP 20] comes out the other end, we've got a clinician with so many more skills, etcetera. So there was a few bit of hard yakka in the middle while we were trying to do without him.” Manager 4b*<br>*“I don't mind investing in… [AH-TRIP because] “we should be getting rid of low value healthcare” Manager 5b*<br>*“It's great to have interested clinicians, but you also need the support of the department and the clinical leads and things to make it doable” HP 2*<br>*“How do you lead a project when you’re not allowed to [take charge and make change]? Yeah, you’re like being careful of stepping on people’s toes.” HP 10*
	Staffing levels and turnover (especially in regional and rural areas)	Barrier	*“I think lots of the team are still concerned about the time requirements to actually run a really good QI project where we don't have a lot of fat within our department to make this happen. So you know when [HP 20] goes, everybody's gotta pick up the slack and do their work for them.” Manager 4b*<br>*“So change of staffing as well. I would say it probably needs to have another refresh to keep it current, because I would say it's less, people would be less knowledgeable about it, some of our less experienced staff in particular.” Manager 1b*<br>*“I was the last man standing” HP 10*
	Visibility and turnover of AH-TRIP champions	Barrier	*“To be honest, I would not know in [site name removed for confidentiality] who else has done it.” HP 20*<br>*“I'm not even sure if [champion role] then got handed on when that dietitian finished up. I don't know if we've got one at the moment.” HP 8*
**i-PARIHS construct**	**Factor**	**Barrier and/or enabler**	**Exemplar quote**

Allied health department/ team (the local context) cont…	Access to local experts, including AH-TRIP WDO, champions and research fellows, health economists	Enabler<br>Barrier	*"But if you ask anybody here, honestly, they would be able to give you some advice." HP 3*<br>*"[Local AH-TRIP WDO] used to come to [workplace] quite a bit and we would talk about the project. So a lot of that support and I would actually then pass on a lot of that information to my [allied health discipline] network." HP 9*<br>*"So I guess people might get confused about "How do [research fellow and AH-TRIP roles] differ? Who do I go to?" For example, we've got a [HP] at the moment doing some research around an assessment tool in [allied health discipline], but he's sort of going to [research fellow], and to be honest, I'm not sure if [research fellow] is aware of AH-TRIP and if or when they could assist or offer anything in terms of them implementing some of that research into practice." Manager 3b*<br>*"I didn't manage to find anyone locally who had that knowledge expertise. And so it was a matter of kind of asking several people and piecing together bits of ideas." HP 17*
Health service (organisational context)	Research culture of the organisation, particularly in regional and rural health services	Barrier	*"[Guidelines/ processes regarding AH-TRIP] are not part of the wider health service either, but again, I think that's because research itself is not necessarily part and parcel of work here." HP 19*<br>*"It sounds terrible that research is not a priority here and realistically, like evidence-based stuff probably isn't a priority either. It's more just getting the job done as much as we can." HP 10*
	Exposure to real-world examples of TRIP	Enabler	*"[The AH-TRIP showcase] was always really valuable and interesting to hear how other people interpreted the TRIP process and how they went through their cycles and how they decided which frameworks to use." HP 1*

i-PARIHS construct	Factor	Barrier and/or enabler	Exemplar quote
Health service (organisational context) cont…	Awareness and involvement in AH-TRIP by others in the multidisciplinary team	Barrier	*"And then I think the rest of the team like nursing, medical, social work etc, I don't know if they've had so much exposure to it. So that's kind of a little bit of a challenge in terms of, you know, making sure our working group moves through the phases, like not just sort of jump into, "Let's put the goal boards up and see how it goes", but let's you know, "Let's follow the steps kind of thing"." HP 13*<br>*"And you know, talking to them about implementation science, they sort of look at you a bit strange 'What are you talking about?' Because a lot of the executive tend to be nurses or medical profession… so it needs to spread to the other professions so that we're all on the same page." HP 9*<br>*"I have brought it up with nursing when they had a different project and just talked about what systems we use and what frameworks and they'd never heard of anything like that." HP 4*
	Project funding timeframes	Barrier	*"To be honest, sometimes we have systems that mean that we have to do it that way. So for example, we might get a grant or some money that has to be spent within the financial year and we're just told we need to spend this money, going 'Go and do something.' And it's often a little bit ill-considered and rushed, but otherwise we'll lose the funding." Manager 3b*
	Ongoing investment beyond the project phase	Barrier	*"The grant was definitely a kick start, but it has needed ongoing commitment clinically…massive in-kind labour costs" HP 5*
	Maturity of the organisation	Barrier	*"I don't think that there's like a lack of awareness around AH-TRIP at [site name] specifically, but I think being able to prioritise it as a big, fledgling organization is a little bit challenging." HP 5*

i-PARIHS construct	Factor	Barrier and/or enabler	Exemplar quote
External to health service (outer context)	COVID-19 pandemic	Barrier	*"At the moment quality improvement is just so challenging with COVID. No clinic, no non-clinical stuff, no non-clinical meetings and all that for so long." HP 18*<br>*"There's been a directive at a higher level to put projects on hold." HP 16*
	Awareness of implementation science broadly	Enabler	*Even just at conferences and things that I'm attending, I'm noticing a lot more around implementation science and knowledge translation. Whereas again, I think 4 or so years ago when I was first delving into this space, it was unheard of, so I think I'm seeing a little bit of a shift in that space, that it's becoming a little bit more common place and understood more broadly." HP 6*
TRIP: Translating Research into Practice (used a synonym for knowledge translation); HP: Health Practitioner participant; WDO: Workforce Development Officer (specific role to support staff education and training).			
